# Contemporary Endodontic Treatment

**DOI:** 10.1155/2012/231362

**Published:** 2012-11-06

**Authors:** Igor Tsesis, Silvio Taschieri, Iris Slutzky-Goldberg

**Affiliations:** ^1^Department of Endodontology, Maurice and Gabriela Goldschleger School of Dental Medicine, Tel Aviv University, Tel Aviv, Israel; ^2^Centre for Research in Oral Health, Department of Biomedical, Surgical, and Dental Sciences, Università degli Studi di Milano and IRCCS Istituto Ortopedico Galeazzi, Milan, Italy; ^3^Department of Endodontics, Hadassah School of Dental Medicine, The Hebrew University of Jerusalem, Jerusalem, Israel

The ultimate goal of the endodontic treatment is to render the root canal system bacteria-free and to prevent the invasion of bacteria and their byproducts from the root canal system into the periradicular tissues.

This special issue presents current research addressing the newest approach to the diagnosis and treatment of the endodontic disease.

Fast development of new materials and procedures gave rise for better preparation and filling techniques and enabled higher standards and better quality of root canal treatment. Innovative approach to the anatomy of the roots allows for the predictable preparation of the root canal system. Evaluation of the latest root canal filling materials and restoration techniques helps elucidate factors responsible for the long-term survival of the endodontically treated teeth. The latest molecular biology techniques may help clarify the composition of microbiota in infected root canals and help in the development of new direction in nonsurgical root canal treatment.

*Igor Tsesis* *Igor Tsesis* *Silvio Taschieri* *Silvio Taschieri* *Iris Slutzky-Goldberg* *Iris Slutzky-Goldberg*

